# Dominance and Prestige Motivations to Lead in Adolescence

**DOI:** 10.1111/jopy.70029

**Published:** 2025-11-20

**Authors:** Jennifer L. Tackett, Cassandra M. Brandes, Kathleen W. Reardon, Allison N. Shields

**Affiliations:** ^1^ Northwestern University Evanston Illinois USA; ^2^ University of Illinois Urbana‐Champaign Champaign Illinois USA; ^3^ Cleveland State University Cleveland Ohio USA; ^4^ Seattle Children's Hospital Seattle Washington USA

**Keywords:** adolescence, construct validity, dominance, leadership, motivations, prestige, youth

## Abstract

**Introduction:**

Dual strategy frameworks of motivation to lead differentiate Dominance motivations, which leverage fear and control to gain power and status, from Prestige motivations, which rely on respect and trust. Substantial research on these motivational pathways has been conducted in adults, but no empirical research studies them earlier in life.

**Methods:**

In a sample of 388 middle adolescents (ages 13–18, both self‐ and mother‐report) and a comparison sample of 563 early adults (ages 18–23), we examined the psychometric properties and personality‐centered nomological network of the Achievement Motivation Scale in this preregistered study.

**Results:**

Results indicated that individual differences in leadership motivations can be reliably assessed in middle adolescence and demonstrate theoretically predicted associations with personality traits. For example, Dominance motivations were associated with higher Extraversion and Social Potency, whereas Prestige motivations were associated with higher Agreeableness and Empathy.

**Discussion:**

These findings suggest that leadership motivations emerge prior to adulthood and are similarly positioned in psychological context across adolescence and early adulthood. Future directions call for more empirical attention to youth leadership and improved measurement of Dominance and Prestige motivations.

*I raise up my voice—not so that I can shout, but so that those without a voice can be heard*.

Yousafzai 2013, Nobel Peace Prize laureate at age 17, teen leader

Despite ample evidence for leadership potential in today's teenagers (Tackett et al. 2023), the vast majority of empirical literature on leadership emergence and development has employed samples of adults (see also Day et al. 2014; Reitan and Stenberg 2019). Understanding the psychology of our world politicians, educators, and CEOs is enormously consequential. Yet, who are these world leaders before they take on these powerful leadership roles? And how do early leadership motivations develop and foster leadership emergence in adolescence?

Leadership motivations are actively studied in adults with a sizable and growing empirical literature. Dual strategy frameworks of adult leadership motivations highlight two distinct pathways to leadership: Dominance and Prestige (Cheng et al. 2010; McClanahan et al. 2022; Maner and Case 2016). Dominance motivations reflect a desire to control resources and gain status through fear and coercion, whereas, Prestige motivations reflect status‐oriented behaviors driven by achieving trust, respect, and consensus from others. The vast literature on these motivations in adults neglects attention to where they come from, how early they emerge, and whether their measurement is generalizable for different age groups, which is the focus of the current study.

Adolescence is a critical period for leadership emergence and development (Tackett et al. 2023). During this developmental period, teens form vocational interests and many will embark on their first employment experience (Eva et al. 2021; Fredricks and Eccles 2010). In addition, today's youth frequently engage in leadership roles—albeit primarily in different contexts (e.g., sports, extracurricular activities, and social justice initiatives; Kelsey and Fuhrman 2020; Wray‐Lake et al. 2022) than those more common for adults. Thus, engagement in the world of emergent leadership is already active for today's teens; however, the scientific literature has thus far left these efforts to proceed with a sparse empirical foundation (Karagianni and Jude Montgomery 2018; Liu et al. 2019; Tackett et al. 2023). The current study seeks to address this critical need for empirical research on leadership‐relevant constructs, such as Dominance and Prestige leadership motivations, in adolescence.

## 1 Motivations to Lead

Motivation to lead is a psychological process central to both leadership potential and behaviors (Chan and Drasgow 2001). Research in adults has suggested that Dominance and Prestige motivations reflect two meaningfully distinct pathways to leadership behaviors (Cheng et al. 2010; McClanahan et al. 2022; Maner and Case 2016). Both Dominance and Prestige reference potential strategies that individuals may employ to increase or maintain their social rank (Maner and Case 2016). Dominance and Prestige strategies differ, however, in their use of fear and coercion versus respect and admiration, respectively, to achieve status goals (Chen et al. 2021; Redhead et al. 2021). They also differ in the extent to which the individual actively regulates status‐seeking behaviors (in the case of Dominance) versus status as conferred by the perceiver (Prestige; McClanahan et al. 2022).

A differential focus on these strategies results in different leadership behaviors. Leaders high in Dominance motivation are more likely to prioritize their own needs over those of the group, whereas the inverse is expected for leaders high in Prestige motivation. These distinct strategies can have an enormous impact on the overall success of the group and other relevant outcomes (Chen et al. 2021), and they are differentially related to basic leadership skills such as promoting leadership based on trust, leading by example, and adaptive communication (Cheng et al. 2010). Thus, as early leadership skills, identifying and understanding individuals' use of these strategies is critical. Researchers in this area have used different measures of Dominance and Prestige motivations and few studies have examined extensive evidence for construct validation of these content scales (see exceptions described below: Körner et al. 2023; Suessenbach et al. 2019). In response to the varying use of different measures and the need to consolidate across the field, a recent review provided the most definitive summary to date of various scales that have been used in the literature and their psychometric properties as applied to adult populations (identifying 67 different scales; Körner et al. 2025).

Personality is differentially associated with Dominance and Prestige strategies, at least in adults (Cheng et al. 2010; Körner et al. 2023; Suessenbach et al. 2019). Dominance motives have been associated with narcissism, aggression, extraversion, and low agreeableness (Cheng et al. 2010; Suessenbach et al. 2019). Prestige motives have been associated with self‐esteem, social acceptance, and conscientiousness, and to some extent narcissism and extraversion (Cheng et al. 2010; Suessenbach et al. 2019). The more extensive and pre‐registered nomological network studies have shown some discrepancies, however—for example, showing mixed evidence for hypothesized associations between agreeableness and Prestige (Körner et al. 2023; Suessenbach et al. 2019).

Peers described individuals rated high in Dominance motivations as less altruistic, cooperative, helpful, ethical, and moral (Cheng et al. 2010). Peers described individuals rated high in Prestige motivations as higher on advice‐giving, more intellectual, and more altruistic, cooperative, helpful, ethical and moral (Cheng et al. 2010). Peers rated both highly dominant and highly prestigious targets as higher in leadership abilities. Another study, also in adults, found that individuals who rated themselves as higher in Dominance also rated themselves as higher in psychopathy and sadism compared to individuals who rated themselves as high in Prestige; whereas, higher narcissism and Machiavellianism were associated with both higher Dominance and Prestige (Blötner et al. 2021). Thus, some evidence was found for both personality overlap and differentiation between the two motivational strategies. Overall, while there is some evidence to draw on for potential personality associations with Dominance and Prestige in adults, the number of studies is small and the overall pattern of findings is still emerging.

It is yet unclear at what developmental stage leadership motivations emerge, or whether these motivations are identical in youth and adults (see Gottfried et al. 2011). Youth social contexts differ quite a bit from those of adults (Lerner et al. 2015; Smetana et al. 2006), and thus, there is reason to anticipate that leadership motivations may present differently at younger ages. However, other individual difference constructs (e.g., FFM personality traits) show clear emergence and developmental growth well before adulthood (De Fruyt et al. 2009; Soto and Tackett 2015), and there is no compelling reason to expect motivations to lead to be different in this regard. The extensive literature using psychometrically sound measures of adolescent personality offers a scientific framework for examining leadership constructs in youth. Taken together, there is reason to expect that leadership motivations emerge before adulthood, but a need for empirical research investigating evidence for this and the extent to which their position in broader psychological space is similar to—or different from—these motivations in adulthood.

Should these motivations exist well before adulthood, we would expect studies of first‐year college students to reflect cumulative developmental processes that have already occurred in childhood and adolescence. Indeed, preliminary evidence suggests that leadership aspirations endorsed by incoming college students already reflect systemic biases in leadership pathways (Bates et al. 2025; Wolniak et al. 2023). Specifically, in a nationally representative sample of US college students, male students endorsed higher leadership aspirations than female students, and the impact of 4 years of college experiences did not alter this difference (Wolniak et al. 2023). At the same time, Black students endorsed higher leadership aspirations than white students at both college entry and exit, suggesting that mechanisms underlying a lack of diversity in who occupies leadership roles are likely complex (Bates et al. 2025). Such work suggests that understanding leadership motivations earlier than adulthood is critical to efforts to diversify the leadership pipeline (e.g., Boatwright and Egidio 2003). Currently, no empirical data exist to document the presence of these specific leadership motivations—Dominance and Prestige—in adolescents or the extent to which they correlate with other indices of early leadership propensity.

## 2 Contextualizing Adolescent Leadership Motivations: A Construct Validation Approach

Construct validation is a fundamental approach to building and refining both theory and measurement of psychological constructs (Clark and Watson 2019; Cronbach and Meehl 1955; Grahek et al. 2021), including adult leadership motivations (Chan and Drasgow 2001; Suessenbach et al. 2019). It is a critical aspect of defining “new” constructs by investigating measurement approaches while also building a broader theoretical framework about what a construct *is* and what it *isn't* (see Credé et al. 2017 for a cautionary tale for researchers who skip this step). Specifically, specification of a nomological network provides a theoretical framework for empirical examination of variables that should be related to the target construct (i.e., convergent validity) and variables that should not be related to the target construct (i.e., divergent validity). Although Dominance and Prestige motivations have not been previously empirically examined in youth, a construct validation approach allows us to draw on the rich empirical literature on adolescent personality, personality pathology, and interpersonal relational functioning in building our hypotheses for adolescent leadership motivations going forward.

Construct validation has been far too neglected in psychological science broadly, and this extends to recent mobilization toward pre‐registration of scientific hypotheses, research questions, and anticipated results. As such, there are not current best practices for pre‐registration of nomological networks, although specification of direction and magnitude of effect sizes is critical for building strong theories (beyond specifying only “positive correlations” which contributes to weak theories; Grahek et al. 2021). In the present study, we pre‐registered a construct validation approach (see related approaches in adult samples in Körner et al. 2023; Suessenbach et al. 2019) to examine evidence for Dominance and Prestige motivations in adolescence that clearly delineates (and, to the extent they are distinct, differentiates) these constructs in a broader nomological network of psychological phenomena.

## 3 The Current Study

The current study contributes the first large empirical investigation of Dominance and Prestige leadership motivations in adolescents. Specifically, it is important to understand whether and how leadership motivations, as they are typically studied in adult samples, manifest in youth (Tackett et al. 2023). Anticipating that individual differences in leadership motivations indeed manifest prior to adulthood, we further examine the nomological network of these motivations—what is the psychological context in which these motivations exist? Construct validation (Brandes et al. 2021; Clark and Watson 2019) is a critical step in interrogating the measurement of constructs of interest, but also in situating these constructs in a broader psychological framework that identifies constructs that are both related and unrelated to the target constructs (i.e., convergent/discriminant validity) and which differentiate leadership motivations (i.e., Dominance and Prestige) from one another. Finally, in working toward a lifespan developmental framework of leadership emergence (Tackett et al. 2023), it is necessary to directly test the extent to which these associations differ from adolescence to emerging adulthood.

In this project, we investigate the psychometric properties and personality‐centered nomological network of a measure of Dominance and Prestige motivations for leadership, the Achievement Motivation Scale (AMS; Cassidy and Lynn 1989), in a sample of community‐based adolescents (*n* = 388) and a comparative sample of early adult university students (*n* = 563). This measure has received no previous validation in youth samples to the best of our knowledge. Specifically, we addressed the following research questions (RQ):

1. Can individual differences in leadership motivations be reliably assessed in adolescence?
2. Do empirical associations support a pre‐registered, theoretically derived, and personality‐centered nomological network for Dominance and Prestige motivations in middle adolescence?
3. Does measurement of Dominance and Prestige motivations, and their associations with personality and related constructs, show differences between this adolescent sample and a sample of emerging adults?

## 4 Transparency and Openness Practices

This project is exploratory in nature; nonetheless, some hypotheses pertaining to the personality‐based nomological network were derived by drawing on similar studies in adults and a broader knowledge base on adolescent personality and social functioning. We therefore preregistered our study rationale, hypotheses tables, and data analytic plan to make clear which aspects of the data analyses were exploratory and which were testing our preplanned hypotheses. The preregistration can be accessed at the project's OSF page (https://osf.io/pbue8). Materials, R syntax, and data output for the analyses and figures can be accessed at the project's OSF page (https://osf.io/ns7qw/). We report how we determined our sample size, all data exclusions, all manipulations, and all measures in the study. Ethics approval was obtained by the Institutional Review Board at Northwestern University for both samples. Deviations from the pre‐registration include analytic models that did not converge properly (split‐half reliabilities, measurement invariance, and profile similarity analyses), and the presentation of key findings and overarching patterns of results to facilitate communication in the present manuscript (comprehensive results and analyses are posted to the OSF page). In addition, given fit indices that did not achieve pre‐registered thresholds, exploratory analyses examining a 3‐factor model are also presented on OSF for further exploration but are not discussed extensively in the current manuscript as they were not pre‐registered.

## 5 Methods

### 5.1 Participants

Sample 1 participants consisted of 388 adolescents (*M*_age_ = 14.94, SD_age_ = 1.51, age range = 13–18 years, 195 female, 193 male) and 348 of their primary caregivers (290 mothers). Race and ethnicity were reported by parents (or by youth if parent‐report data were unavailable) as follows: 61.60% white/Caucasian, 10.57% Black/African American, 9.79% Asian/Asian American, 6.70% Latin American, and 10.82% other/multiple races. One participant (0.52%) did not report race/ethnicity. Adolescents were recruited from a large urban area in the midwestern United States via flyers distributed online, in local high schools, and at community events. Inclusion criteria were English fluency and age range between 13 and 17 years old; 18‐year‐olds were also included if they were still enrolled in high school. Exclusion criteria were the presence of intellectual disability, neurodevelopmental disorders, or psychotic disorders in the adolescents.

Sample 2 participants consisted of 563 young adults (*M*_age_ = 18.93, SD_age_ = 1.01, age range = 18–23, 316 female, 241 male, 4 non‐binary, 2 did not report). Race/ethnicity was reported by the young adults as follows: 33.21% white/Caucasian, 8.17% Black/African American, 29.13% Asian, 8.53% Latin American, 19.89% multiple or other races, and 1.07% did not report on their race/ethnicity. Young adults were recruited via an introductory psychology course at a private midwestern university. Inclusion criteria were young adults 18 years of age and older and English fluency. Exclusion criteria included previous diagnoses of intellectual disability, neurodevelopmental disorders, and/or psychotic disorders in the young adults.

### 5.2 Measures

#### 5.2.1 Achievement Motivation Scale (Cassidy and Lynn 1989)

Fourteen items were drawn from the AMS to measure an individual's orientation toward Dominance and Prestige,^1^ consistent with the approach taken by Maner and Mead (2010) and others (see Körner et al. 2025 for a review). The Dominance scale was measured with seven items that assess an individual's desire for power and authority (e.g., “I think I would enjoy having authority over other people”). The Prestige scale was measured with seven items that assess an individual's desire for respect and admiration (e.g., “I would like an important job where people look up to me”). Raters used a 2‐point *Yes*/*No* scale for all items, consistent with the original response format used for the AMS. Coefficient alphas for the AMS scales in the present study ranged from 0.77 to 0.93. The AMS was completed by parents and self in Sample 1 and self in Sample 2.

#### 5.2.2 Big Five Inventory‐2 (BFI‐2; Soto and John 2017)

The BFI‐2 is a 60‐item self‐report questionnaire that assesses the Five Factor Model (FFM) of personality and 15 facets. Items on the BFI‐2 include short, descriptive phrases that assess several characteristics of an individual (e.g., “I am someone who is compassionate, has a soft heart”). Raters used a 5‐point Likert‐type scale ranging from (1) *disagree strongly* to (5) *agree strongly*. Coefficient alphas for the BFI‐2 personality scales in the present study ranged from 0.78 to 0.92. The BFI‐2 was completed by youth and parents in Sample 1 and young adults in Sample 2.

#### 5.2.3 Dimensional Personality Symptom Itempool (DIPSI; De Clercq et al. 2006)

The DIPSI is a questionnaire that assesses personality pathology traits. The DIPSI consists of four higher‐order dimensions of personality pathology: Disagreeableness, Emotional Instability, Introversion, and Compulsivity. Participants rated each item using a 5‐point Likert‐type scale ranging from (1) not characteristic to (5) highly characteristic. Parents rated their children using the full 172‐item version of the DIPSI, the full version of the scale; youth self‐reported their personality pathology traits using six selected facets from the original Disagreeableness domain (45 items total); young adults self‐reported their personality pathology traits using the 81‐item DIPSI‐Short Form (Reardon and Tackett 2018). Coefficient alphas for the DIPSI scales in the present study ranged from 0.68 to 0.95.

#### 5.2.4 Inventory of Children's Individual Differences‐Short (ICID‐S; Deal et al. 2007; Halverson et al. 2003)

The ICID‐S is a 50‐item questionnaire that assesses children's personality traits across the FFM model. Items in the ICID‐S include short, descriptive phrases that assess a child's thoughts (e.g., “I am thoughtful of others”), feelings (e.g., “I am insecure”), and behaviors (e.g., “I am a hard worker”). Youth rated each item using a 7‐point Likert‐type scale ranging from (1) *much less than the average child* to (7) *much more than the average child*. Coefficient alphas for the ICID‐S personality scales in the present study ranged from 0.72 to 0.87. The ICID‐S was completed by youth in Sample 1.

#### 5.2.5 Multidimensional Personality Questionnaire (MPQ; Tellegen 1982)

The MPQ is an omnibus measure of normal‐range personality with a higher and lower‐order structure. Three lower‐order scales from the MPQ, comprised of 57 self‐report items were administered to expand coverage of the personality trait space beyond that typically assessed with FFM measures: Social Potency, Traditionalism, and Absorption. Raters rated each item using a 4‐point Likert‐type scale ranging from (1) definitely false to (4) definitely true. Coefficient alphas for the MPQ scales in the present study ranged from 0.79 to 0.88. The MPQ was completed by youth in Sample 1 and young adults in Sample 2.

#### 5.2.6 Interpersonal Reactivity Index (IRI; Davis 1980)

The IRI is a 28‐item questionnaire that assesses an individual's empathic tendencies using four scales: Fantasy, Perspective‐Taking, Empathic Concern, and Personal Distress. Raters rated each item using a 5‐point Likert‐type scale ranging from (1) *does not describe me* to (5) *describes me very well*. Coefficient alphas for the IRI scales in the present study ranged from 0.71 to 0.84. The IRI was completed by youth and caregivers in Sample 1 only.

### 5.3 Procedure

#### 5.3.1 Sample 1

Data from Sample 1 was collected as part of the Game Changers project, a broad study investigating numerous domains including personality, leadership, and relationships. Caregivers provided consent and youth provided verbal assent to participate in the study. Youth were taken to a separate testing room within the lab and were asked to complete online questionnaires, including the AMS, BFI‐2, DIPSI‐45, ICID‐S. Caregivers also completed online questionnaires pertaining to their children. Families received reimbursement for local travel to the lab, and both caregivers and youth were given monetary compensation (gift cards) for participation in the overall extended protocol.

#### 5.3.2 Sample 2

Young adults were asked to participate in a lab‐based survey study on youth leadership. Young adults were asked to arrive at the lab and complete a variety of questionnaires, including the AMS, BFI‐2, DIPSI‐SF, DPS, and MPQ. Participants were compensated with course credit for participation.

## 6 Results

### 6.1 RQ1: Can Individual Differences in Leadership Motivations Be Reliably Assessed in Adolescence?

In the adolescent sample (Sample 1), Cronbach's alpha was 0.84 for self‐report Dominance, 0.75 for self‐report Prestige, 0.89 for parent‐report Dominance, and 0.81 for parent‐report Prestige. The average interitem correlation (AIC) in Sample 1 was 0.43 for self‐report Dominance, 0.29 for self‐report Prestige, 0.55 for parent‐report Dominance, and 0.37 for parent‐report Prestige. Taken together, internal consistency ranged from adequate to good across reporters and AIC estimates largely fell within a desirable range for this breadth of construct (e.g., 0.15–0.50; Clark and Watson 2019). Interrater reliability for parent‐ and youth‐report Dominance was *r* = 0.403 (*n* = 291; *p* < 0.001), and for parent‐ and youth‐report Prestige was *r* = 0.204 (*n* = 291; *p* < 0.001). Overall, these findings suggest that individual variability in Dominance and Prestige motivations can be reliably measured in adolescence, with moderate evidence for agreement across informants.

As pre‐registered, we examined structural validity by using confirmatory factor analysis (CFA) using tetrachoric correlations and weighted least squares—mean and variance adjusted (WLSMV). The fit of the pre‐registered 2‐factor model was poor by virtually all metrics in all three datasets (parent‐ and self‐report from Sample 1, self‐report from Sample 2). Specifically, CFI (range 0.804–0.899) and TLI (range 0.765–0.879) did not meet our threshold of 0.95, RMSEA (range 0.065–0.136) did not fall ≤ 0.05, and SRMR (range 0.081–0.120) did not fall ≤ 0.06. Thus, we did not pursue additional structural analyses or measurement invariance analyses but flagged this as a clear need for further examination of the best measures for these constructs. Full results from the pre‐registered structural validity analyses can be found on the OSF page for this project (https://osf.io/ns7qw/?view_only=37bb03aa42e74666b0a862978b68e988). Taken together, the reliability estimates indicate general support for individual differences in leadership motivations in adolescence and do not indicate substantial reliability differences between adolescents and early adults, although the version of the AMS used in the present study does not indicate good evidence for structural validity in either age group. Moving forward, we used summed scores for the scales to maintain consistency with previous research but noted the need for further research to interrogate the best measurement of these constructs using items from the AMS scale.

### 6.2 RQ2: Do Empirical Associations Support a Pre‐Registered, Theoretically Derived Personality‐Centered Nomological Network for Dominance and Prestige Motivations in Middle Adolescence?

**FIGURE 1:**
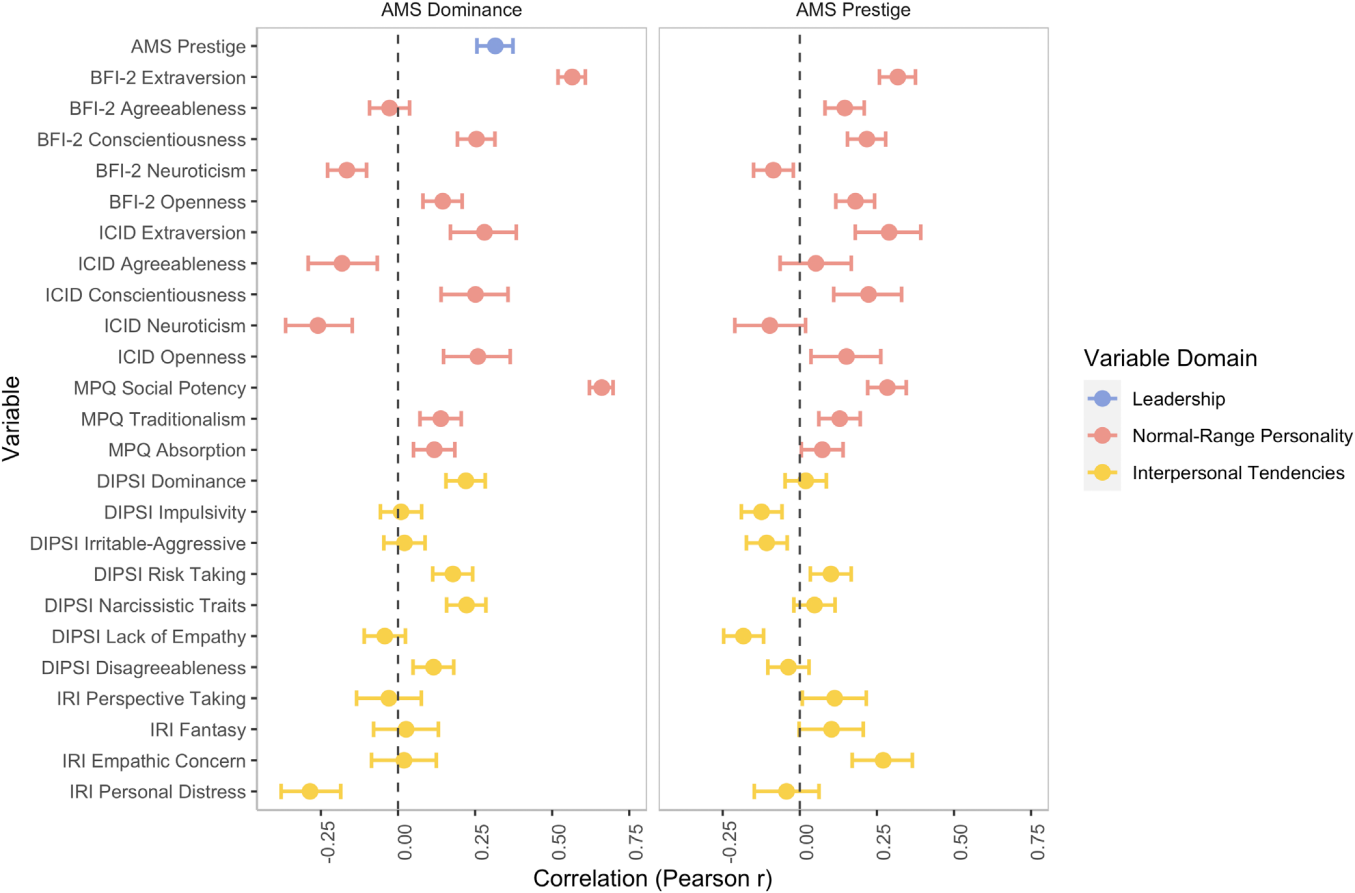
Convergent/divergent correlations with Dominance and Prestige.

Pearson correlations between Dominance and Prestige motivations in Sample 1 were examined relative to the pre‐registered nomological network (both full correlational results and the pre‐registered nomological network can be found online at https://osf.io/ns7qw/?view_only=37bb03aa42e74666b0a862978b68e988). Associations are presented for youth self‐report data (see Figure 1) given that parents did not complete identical measures to the youth and predictions did not differ by informant. Tables 1 and 2 present the pre‐registered effect size (magnitude and direction, denoted by a “Y”) along with the estimated effect size in this sample.

**TABLE 1:** Convergent/discriminant validity predictions for AMS Dominance.

Construct	[< −0.30]	[−0.30, −0.21]	[−0.20, −0.11]	[−0.10, 0.10]	[0.11, 0.20]	[0.21, 0.30]	[> 0.30]
BFI Neuroticism		Y	−0.17				
BFI Extraversion						Y	0.54
BFI Conscientiousness					Y	0.23	
BFI Agreeableness		Y		NS			
BFI Openness to Experience				Y; NS			
ICID Neuroticism		Y; −0.26					
ICID Extraversion					Y; 0.28		
ICID Conscientiousness					Y	0.25	
ICID Agreeableness		Y	−0.18				
ICID Openness to Experience					Y	0.26	
MPQ Social Potency						Y; 0.22	0.65
MPQ Traditionalism				Y; NS			
MPQ Absorption				Y; NS			
DIPSI Disagreeableness					Y; 0.12		
DIPSI Dominance‐Egocentrism						Y; 0.22	
DIPSI Impulsivity			Y	NS			
DIPSI Irritable‐Aggressive				NS	Y		
DIPSI Risk Taking					Y; 0.18		
DIPSI Narcissistic Traits						Y; 0.22	
DIPSI Lack of Empathy				NS		Y	
IRI Perspective Taking			Y	NS			
IRI Empathic Concern		Y		NS			
IRI Fantasy				Y; NS			
IRI Personal Distress		Y; −0.29					

*Note:* Predictions (denoted Y) correspond to preregistered hypothesized effects; numbers indicate observed effect in the present study (Pearson correlation coefficients). All relationships are within informant; all reported correlations significant at *p* < 0.05.

Abbreviations: AMS, Achievement Motivation Scale; BFI, Big Five Inventory‐2; DIPSI, Dimensional Symptom Itempool for Children; ICID, Inventory of Children's Individual Differences; IRI, Interpersonal Reactivity Index; MPQ, Multidimensional Personality Questionnaire; NS, no significant relationship.

**TABLE 2:** Convergent/discriminant validity predictions for AMS Prestige.

Construct	[< −0.30]	[−0.30, −0.21]	[−0.20, −0.11]	[−0.10, 0.10]	[0.11, 0.20]	[0.21, 0.30]	[> 0.30]
BFI Neuroticism				Y; NS			
BFI Extraversion					Y		0.31
BFI Conscientiousness						Y; 0.22	
BFI Agreeableness					0.20	Y	
BFI Openness to Experience					Y; 0.14		
ICID Neuroticism				Y; NS			
ICID Extraversion					Y	0.29	
ICID Conscientiousness						Y; 0.22	
ICID Agreeableness				NS		Y	
ICID Openness to Experience					Y; 0.15		
MPQ Social Potency						Y; 0.28	
MPQ Traditionalism			Y		0.21		
MPQ Absorption				Y; NS			
DIPSI Disagreeableness			Y	NS			
DIPSI Dominance‐Egocentrism				Y; NS			
DIPSI Impulsivity			Y; −0.12				
DIPSI Irritable‐Aggressive		Y	−0.11				
DIPSI Risk Taking				0.10	Y		
DIPSI Narcissistic Traits				Y; NS			
DIPSI Lack of Empathy		Y	−0.18				
IRI Perspective Taking					0.11	Y	
IRI Empathic Concern						Y; 0.27	
IRI Fantasy				Y; NS			
IRI Personal Distress				NS	Y		

*Note:* Predictions (denoted Y) correspond to preregistered hypothesized effects; Numbers indicate observed effects in the present study (Pearson correlation coefficients). All relationships are within informant; all reported correlations significant at *p* < 0.05.

Abbreviations: AMS, Achievement Motivation Scale; BFI, Big Five Inventory‐2; DIPSI, Dimensional Symptom Itempool for Children; ICID, Inventory of Children's Individual Differences; IRI, Interpersonal Reactivity Index; MPQ, Multidimensional Personality Questionnaire; NS, no significant relationship.

Of the 24 pre‐registered effect sizes for associations with Dominance, 21 (87.5%) were in the hypothesized direction and 14 (58%) were equal to or greater than the hypothesized magnitude. Of the 24 pre‐registered effect sizes for associations with Prestige, 21 (87.5%) were in the hypothesized direction and 18 (75%) were equal to or greater than the hypothesized magnitude. Given the lack of direct empirical evidence informing these associations, these results demonstrate substantial support for the pre‐registered predictions for the nomological networks of adolescent Dominance and Prestige based on theory and evidence in adult samples.

In Sample 1, correlations between Dominance and Prestige subscales were moderate (*r* = 0.30, 95% CI [0.20, 0.39]). In terms of over normal‐range personality associations, Dominance was characterized most strongly by high BFI Extraversion (*r* = 0.54; also for mother‐report, *r* = 0.60) and high MPQ Social Potency (*r* = 0.65). Medium to large effect sizes were also found for associations between Dominance and high ICID Extraversion (*r* = 0.28), high ICID Openness to Experience (*r* = 0.26), low ICID Neuroticism (*r* = −0.26), high ICID Conscientiousness (*r* = 0.25), and high BFI Conscientiousness (*r* = 0.23; also for mother‐report, *r* = 0.26). Overall effect sizes for associations with Prestige were not as strong, with the strongest effects emerging for high BFI Extraversion (*r* = 0.31; also for mother‐report, *r* = 0.45), ICID Extraversion (*r* = 0.29), MPQ Social Potency (*r* = 0.28), BFI Conscientiousness (*r* = 0.22; also for mother‐report, *r* = 0.41), ICID Conscientiousness (*r* = 0.22), MPQ Traditionalism (*r* = 0.21), and BFI Agreeableness (*r* = 0.20; also for mother‐report, *r* = 0.30).

Regarding associations with self‐reported personality pathology domains, Dominance was associated with DIPSI Dominance‐Egocentrism traits (*r* = 0.38; also for mother‐report, *r* = 0.30) and to some extent DIPSI Narcissistic Traits (*r* = 0.17; also for mother‐report, *r* = 0.27). Prestige was associated with lower DIPSI Lack of Empathy (*r* = −0.30; also for mother‐report, *r* = −0.26) and lower DIPSI Irritable‐Aggressive (*r* = −0.16; also for mother‐report, *r* = −0.21) and Impulsivity traits (*r* = −0.16; also for mother‐report, *r* = −0.29). Finally, associations with interpersonal relationship scales showed Dominance to be primarily associated with low levels of self‐reported IRI (empathic) Personal Distress (*r* = −0.29; not as strong in mother‐report, *r* = −0.08); whereas Prestige was primarily associated with high levels of IRI empathic concern (*r* = 0.27; not as strong in mother‐report, *r* = 0.06).

Absolute values were calculated to examine those associations showing the largest and smallest difference between Dominance and Prestige for further description of content captured by these subscales (see Table 3). The sharpest distinction between Dominance and Prestige motivations indicated that Dominance was marked by higher levels of Dominance/Egocentric traits and Social Potency, while Prestige was associated with higher Agreeableness and Empathy. All of the highest differential traits were significantly different according to Steiger's (1980) test for comparing correlation size (all converted Fishers *r*‐to‐*z*—were ≥ 3.849 and all associated *p‐*values were < 0.000).

**TABLE 3:** Absolute differences between associations for AMS Dominance and AMS Prestige.

Construct	Dominance	Prestige	Absolute difference
MPQ Social Potency	0.65	0.28	0.37
DIPSI Dominance‐Egocentrism	0.38	0.02	0.36
BFI Agreeableness	−0.07	0.20	0.27
DIPSI Lack of Empathy	−0.03	−0.30	0.27
DIPSI Disagreeableness	0.13	−0.13	0.25
IRI Fantasy	0.02	0.27	0.25
IRI Personal Distress	−0.29	−0.04	0.24
BFI Extraversion	0.54	0.31	0.23
ICID Agreeableness	−0.18	0.05	0.23
ICID Neuroticism	−0.26	−0.10	0.16
IRI Perspective Taking	−0.03	0.11	0.14
DIPSI Impulsivity	−0.03	−0.16	0.13
DIPSI Narcissistic Traits	0.17	0.05	0.12
ICID Openness to Experience	0.26	0.15	0.11
DIPSI Risk Taking	0.08	−0.04	0.11
MPQ Traditionalism	0.11	0.21	0.10
BFI Neuroticism	−0.17	−0.08	0.09
IRI Empathic Concern	0.03	0.10	0.08
DIPSI Irritable‐Aggressive	−0.10	−0.16	0.06
ICID Conscientiousness	0.25	0.22	0.03
BFI Openness to Experience	0.12	0.14	0.02
BFI Conscientiousness	0.23	0.22	0.01
ICID Extraversion	0.28	0.29	0.01
MPQ Absorption	0.06	0.05	0.01

*Note:* Constructs are ordered from largest to smallest absolute difference.

Abbreviations: AMS, Achievement Motivation Scale; BFI, Big Five Inventory‐2; DIPSI, Dimensional Symptom Itempool for Children; ICID, Inventory of Children's Individual Differences; IRI, Interpersonal Reactivity Index; MPQ, Multidimensional Personality Questionnaire.

### 6.3 RQ3: Does Endorsement of Dominance and Prestige Motivations, and Their Associations With Personality and Related Constructs, Show Differences Between This Adolescent Sample and a Sample of Emerging Adults?

Overall findings for reliability and structural validity in the emerging adulthood sample (Sample 2) were largely similar to those for the adolescent sample (Sample 1). In Sample 2, Cronbach's alpha was 0.87 (compared to 0.84 in Sample 1) for self‐report Dominance and 0.82 (compared to 0.75) for self‐report Prestige. The AIC in Sample 2 was 0.49 (compared to 0.43 in Sample 1) for self‐report Dominance and 0.39 (compared to 0.29) for self‐report Prestige.

In terms of comparing the nomological networks between adolescent and EA samples, results were strikingly similar. Full results can be viewed and compared at our OSF page, including analyses run on the pooled sample of self‐report data from both Samples 1 and 2 self‐reports (https://osf.io/ns7qw/?view_only=37bb03aa42e74666b0a862978b68e988). In Sample 2, correlations between Dominance and Prestige subscales were moderate (*r* = 0.33, 95% CI [0.25, 0.40], compared to *r* = 0.30, 95% CI [0.20, 0.39] in Sample 1). In terms of overall findings, for normal‐range personality associations in Sample 2.

Dominance was once again characterized most strongly by high BFI Extraversion (*r* = 0.58, 95% CI [0.52, 0.63], compared to *r* = 0.54, 95% CI [0.46, 0.61] in Sample 1) and high MPQ Social Potency (*r* = 0.67, 95% CI [0.62, 0.71], compared to *r* = 0.65, 95% CI [0.57, 0.71] in Sample 1). A medium to large effect size was also found for associations between Dominance and high BFI Conscientiousness (*r* = 0.27, 95% CI [0.19, 0.34], compared to *r* = 0.23, 95% CI [0.13, 0.33] in Sample 1; the ICID was not administered to Sample 2). Overall effect sizes for associations with Prestige were again not as strong, with the strongest effects emerging for BFI Extraversion (*r* = 0.33, 95% CI [0.25, 0.40], compared to *r* = 0.31, 95% CI [0.21, 0.40] in Sample 1), MPQ Social Potency (*r* = 0.29, 95% CI [0.21, 0.37], compared to *r* = 0.28, 95% CI [0.17, 0.38] in Sample 1), and BFI Conscientiousness (*r* = 0.21, 95% CI [0.13, 0.29], compared to *r* = 0.22, 95% CI [0.12, 0.32] in Sample 1). Slight differences in magnitude emerged for both MPQ Traditionalism (*r* = 0.10, 95% CI [0.01, 0.18] in Sample 2, *r* = 0.21, 95% CI [0.10, 0.32] in Sample 1), and BFI Agreeableness (*r* = 0.10, 95% CI [0.02, 0.18] in Sample 2, *r* = 0.20, 95% CI [0.09, 0.29] in Sample 1), although these differences were not statistically significant (Fisher's *z* = 1.517, *p* = 0.129; *z* = 1.498, *p* = 0.134).

Overall associations with self‐reported personality pathology domains were smaller in magnitude and smaller in terms of absolute effect size differences between Samples 1 and 2. Dominance in Sample 2 showed a small to medium association with DIPSI Dominance–Egocentrism traits (*r* = 0.17, 95% CI [0.08, 0.25], compared to *r* = 0.38, 95% CI [0.28, 0.48] in Sample 1), DIPSI Narcissistic Traits (*r* = 0.25, 95% CI [0.17, 0.33], compared to *r* = 0.17, 95% CI [0.06, 0.28] in Sample 1) and DIPSI Risk‐Taking traits (*r* = 0.23, 95% CI [0.15, 0.31], compared to *r* = 0.08, 95% CI [−0.04, 0.19] in Sample 1). Prestige showed small associations with lower DIPSI Lack of Empathy (*r* = −0.14, 95% CI [−0.22, −0.06], compared to *r* = −0.30, 95% CI [−0.40, −0.19] in Sample 1) and higher DIPSI Risk‐Taking traits (*r* = 0.15, 95% CI [0.06, 0.23], compared to *r* = −0.04, 95% CI [−0.15, 0.08] in Sample 1). Overall associations with personality pathology traits were small with some discrepancies across studies; thus differential patterns in particular should be interpreted cautiously. Taken together, however, these results indicate that the measurement and construct validity of Dominance and Prestige are largely consistent across adolescence and emerging adulthood.

## 7 Discussion

The current study presents the first empirical examination of Dominance and Prestige leadership motivations in a sample of 388 adolescents (ages 13–18) and a comparison sample of 563 emerging adults (ages 18–23). This study supports the presence and measurement of individual differences in these well‐documented motivations toward leadership pathways well before adulthood, where they have exclusively been studied to date. This study further provides evidence for a theoretically meaningful and pre‐registered nomological network for both Dominance and Prestige motivations in this adolescent sample. Finally, these data suggest little differentiation in either the measurement or the broader nomological networks of Dominance and Prestige when comparing middle adolescence to emerging adulthood, paving the way for a greater understanding of the development of leadership motivations across the lifespan.

Use of the AMS to measure Dominance and Prestige is long‐standing in this area of research (Maner and Mead 2010). Although the AMS showed adequate reliability in this adolescent sample (internal consistency, inter‐item correlations, and cross‐informant correlations), structural validity analyses indicated potential measurement problems, particularly with the Prestige domain. These strengths and weaknesses were mirrored in the emerging adult sample, indicating that the problems may not be due to the use of an adult measure in a younger age group. Correlations between adolescent self‐report and mother‐report indicated better convergence for Dominance than Prestige. Mother‐reported associations between Dominance and Prestige motivations with personality and personality pathology traits largely mirrored those for youth self‐report, although stronger associations were found for youth self‐reported factors of empathy (which parents may have less observable exposure to).

Indeed, more recent research on this topic has produced newer measures of Dominance and Prestige that may perform better (e.g., Cheng et al. 2010; Körner et al. 2023; Suessenbach et al. 2019). More importantly, while preparing the current manuscript, a comprehensive systematic literature review was published examining these measurement questions (albeit in studies with adult participants; Körner et al. 2025). These researchers highlight concerns with the many different measures currently used in this field and the lack of comprehensive validation studies, and further provide helpful suggestions for future research on these constructs. Beyond basic measurement concerns, the authors further caution against issues of jingle‐jangle wherein researchers may use the same term to refer to substantially different concepts and different terms to refer to the same concepts. Given the relative newness of investigating these constructs in youth, it will be similarly important for future research to attend to differences from status, popularity, power, and other relevant constructs commonly studied in developmental science (Tackett et al. 2023).

Given these mixed findings for the scale properties of the measure used here, examinations of the nomological network relied on summed scores of the scales as they have been published and used in the literature to facilitate comparability to previous research using this measure in adult samples. Overall, most of our pre‐registered predictions in the nomological network were supported. Over 80% of the effect sizes were in the predicted direction and over half were at the predicted magnitude or stronger. Interestingly, the largest effects were found for the Dominance network, although a greater number of the predicted effects for Prestige met or exceeded the predicted magnitude. Although pre‐registration of construct validation efforts is still in its infancy, we encourage researchers to pre‐register both the direction and magnitude of effect sizes and consider methods to evaluate the overall “strength” of a nomological network such as the one presented in the current manuscript.

In terms of the normal‐range personality associations, evidence for both similarities and differences between Dominance and Prestige emerged, which we predicted in our pre‐registration and is supported by the adult literature (Cheng et al. 2010; Körner et al. 2023; Suessenbach et al. 2019). Although both Dominance and Prestige were associated with high levels of Extraversion and Social Potency, these effects were particularly pronounced for Dominance motivations to the extent that they seem to represent a psychological core that is much more central to Dominance motivations than Prestige motivations. Both showed modest associations with higher Conscientiousness. The clearest evidence for divergence was the association between higher Agreeableness and Prestige; whereas the association with Dominance was smaller than that with Prestige and also in the opposite direction (lower Agreeableness). Results also suggested higher levels of Traditionalism associated with Prestige and lower levels of Neuroticism associated with Dominance, but these smaller effects should be interpreted cautiously.

Findings for associations with personality pathology traits and interpersonal empathy were less consistent with predictions, although those that did emerge were largely theoretically consistent with our pre‐registered predictions and the broader literature. In addition, in contrast to the association with normal‐range personality traits, these associations emphasized differences between Dominance and Prestige more than similarities. Dominance motivations were particularly associated with higher Dominance/Egocentrism traits from the DIPSI and lower levels of empathic Personal Distress, with more modest associations with higher Narcissistic and Disagreeable traits. Prestige, on the other hand, was associated with lower levels of Lack of Empathy traits and higher levels of Empathic Concern. Overall, these associations with higher narcissism (Dominance) versus higher empathy (Prestige) similarly mirror previous findings in research with adults (Cheng et al. 2010; Körner et al. 2023; Suessenbach et al. 2019).

Taken together, we see normal‐range personality traits such as high Extraversion/Social Potency and somewhat higher Conscientiousness perhaps reflecting general endorsement of motivations to lead, while traits such as agreeableness (high for Prestige, low for Dominance), empathy (high for Prestige, low for Dominance), narcissism (higher for Dominance), and maladaptive social dominance (higher for Dominance) offer psychological context for how these motivational pathways are distinct even in middle adolescence. This work extends previous investigations of the nomological network surrounding adult leadership motivations (Chan and Drasgow 2001; Suessenbach et al. 2019) and offers the first empirical application in adolescence. It is notable that high Social Potency (social dominance) and high Conscientiousness were highlighted as likely personality trait precursors for youth leadership in a recent theoretical review (Tackett et al. 2023). The authors also highlighted the frequent omission of social potency/social dominance content in measures of youth personality, which limits the use of common measures to identify early leadership propensity and calls for more expansive and attentive measurement approaches in this domain.

We also investigated whether substantive differences in the nomological network would emerge for this younger middle adolescent sample versus emerging adults. Although we did not predict such differences, there were two important reasons for testing this question: (1) one potential “reason” for the lack of research on leadership motivations in adolescence is that they are seen as only present in and consequential for adult populations, and (2) work that bridges developmental epochs is critical to build cumulative knowledge toward a lifespan developmental understanding of leadership constructs. Indeed, as expected, results for the emerging adult sample were largely indistinguishable from the adolescent sample. In general, the measure of Dominance and Prestige functioned similarly in both age groups and nomological networks were largely consistent, even despite the different populations the samples were drawn from (community adolescents vs. undergraduate students at a private institution). Thus, the present findings support the idea that leadership motivations are not only present before adulthood, but they are positioned in a highly similar psychological context across adolescence to emerging adulthood.

The importance of understanding early motivational pathways to leadership cannot be overstated. Leadership is critically consequential for societal functioning and outcomes, but different leadership styles and pathways can have very different results. Dominance motivated leadership is associated with numerous negative outcomes including self‐serving behaviors, increased punishment of subordinates, and decreased autonomy for followers (Cheng et al. 2010; Maner and Case 2016; Van Kleef et al. 2021). Societal leadership needs are vast and complex, and best served by identifying and fostering leadership skills in our youth. As one example, political polarization has been surging in recent years, largely due to increasing “out‐party hate” (Finkel et al. 2020), a challenge likely to respond quite differently to Dominance versus Prestige leadership styles. Other increasing challenges facing the world, such as those embodied by the United Nations Sustainable Development Goals (UN DESA 2023), require immediate and mobilizing leadership resources that resolve conflict rather than create it. Our greatest leadership resource is in our youth, the leaders of tomorrow—we must better understand how to identify and develop the future leaders that the world needs.

### 7.1 Limitations, Constraints on Generality, and Future Directions

One clear limitation of findings presented in the current study is, as mentioned, the weakness in the measure used of Dominance and Prestige motivations. One advantage of transparency is commitment to the research questions in advance, which highlights retrospective decisions one might make differently. Using a long‐standing measure in the field was a choice made to increase direct comparability with the broader adult literature, but that restricted inclusion of more updated measures of Dominance and Prestige motivations (Körner et al. 2023; Suessenbach et al. 2019) and recent psychometric interrogations of measurement of these constructs (Körner et al. 2025). Further work will be needed to understand how these newer measures perform cross‐culturally (both of the cited studies were conducted with German adults) and across different age groups. A related point is that neither the adolescent or emerging adult samples were representative of the populations from which they were sampled; future research should sample participants much more broadly to investigate generalizability of these findings in younger age groups. Another interesting area for further investigation would be a more focused study of psychometric properties of Dominance and Prestige scales at the item level; it may be that item function and information changes across age or in reference to different external correlates. Despite these limitations, the current data nonetheless underscore the presence of these constructs as meaningful individual differences in adolescence and offer a starting point to build an incremental and methodologically improved research base.

Future research should increase methodological innovation in additional ways (see Liu et al. 2021; Lord et al. 2017). Self‐reported leadership motivations are not the same as the conferral of leadership status by others or the actual effectiveness of leadership behaviors. Future research should examine perceptual and cross‐informant measurement of leadership potential and effectiveness and also incorporate objective measures of leadership effectiveness to evaluate criterion validity. Another measurement area of focus is seen in the discrepant findings for FFM personality traits from divergent measures in this study (Soto and Tackett 2015). Childhood personality measures (i.e., ICID) were largely developed using parent and teacher free descriptors of their children, whereas adult personality measures (i.e., BFI) traditionally arose from dictionary language analysis of human traits (and, of course, were normed on distinct age groups). These findings illustrate how trait content can vary from measure to measure, even when broadly mapping on to a shared framework such as the FFM (see also Tackett et al. 2013, for an illustration of this effect across child personality and temperament measures). Although it is not always practical or feasible to incorporate more than one personality measure in any given study, this variability emphasizes the need to replicate findings across multiple measures and calls on researchers to better understand the actual item‐level content in the measures they are using.

Research on Dominance and Prestige motivations and strategies is increasingly looking to the social context (e.g., Van Kleef et al. 2021) and broader cultural context (e.g., Torelli et al. 2020) to understand when these motivations emerge and under what conditions they are effective. This work indicates that certain situational features (e.g., competition over collaboration) or cultural factors (e.g., power over egalitarianism) may pull for Dominance strategies over Prestige. The samples here do not allow examination of either proximal or distal contextual factors, which should be examined in future research and more diverse samples. Additionally, as each younger generation becomes increasingly more globalized than the next (McKenzie 2019), it will be especially important to understand how sociocultural context impacts leadership motivations and their effects on leadership and status conferral.

### 7.2 Summary

In conclusion, the present study represents the first empirical investigation of Dominance and Prestige leadership motivations in a sample of early to middle adolescents. These findings suggest that nuanced motivations to lead do in fact exist well before adulthood, where they have exclusively been studied, and can be contextualized in a much richer backdrop of substantive psychological constructs. Findings support some aspects reflecting overall motivations to lead, including high extraversion and conscientiousness. Evidence also supported differentiation between Dominance and Prestige motivations, with Dominance more associated with social Dominance, low agreeableness, and low empathy, while Prestige was more associated with high agreeableness and high empathy. Comparisons between our middle adolescent sample and a sample of emerging adults suggest that Dominance and Prestige motivations do not show remarkable differences between these two age groups, supporting continuity for a lifespan development model of leadership that emerges much earlier than adulthood. The need and opportunity to better identify and develop the next generation of leaders is critical but will only be achieved with increased scientific attention to this grossly neglected area of study.

## Author Contributions

J.L.T., C.M.B., and K.W.R. conceived the study, J.L.T., C.M.B., K.W.R., and A.N.S. conducted data collection, data analysis, writing of the manuscript, and manuscript revisions.

## Endnotes

^1^
Dominance items: “I think this participant would enjoy having authority over other people,” “If given the chance, this participant would make a good leader of people,” “I think this participant is usually a leader in their group,” “This participant enjoys planning things and deciding what other people should do,” “This participant likes to give orders and get things going,” “People take notice of what the participant says,” “When a group this participant belongs to plans an activity, they would rather direct it than help out and have someone else organize it;” Prestige items: “This participant would like an important job where people looked up to them,” “The participant likes talking to people who are important,” “The participant wants to be an important person in the community,” “The participant really admires people who have fought their way to the top,” “If the participant had enough money they would not work,” “Even if they won a great deal of money on the pools, the participant would prefer to continue to work,” “If unemployment benefits were really high this participant would still prefer to work.”
